# Stimulated Raman Spectroscopy for Intraoperative Glioblastoma Diagnosis—A Complementary Tool to Frozen Section?

**DOI:** 10.3390/cancers18071053

**Published:** 2026-03-24

**Authors:** Christoph Sippl, Felix Stark, K. Isabel Schneider, Bernardo Reyes Medina, Walter Schulz-Schaeffer, Maximilian Brinkmann, Felix Neumann, Ramon Droop, Steffen Ullmann, Thomas Würthwein, Tim Hellwig, Lucas Hoffmann, Nathan Monfroy, Fatemeh Khafaji, Safwan Saffour, Karim Gaber, Stefan Linsler

**Affiliations:** 1 Department of Neurosurgery, Medical Campus Upper Franconia/Bayreuth, Friedrich Alexander University, 95445 Bayreuth, Germany; felix.sta.stark@fau.de (F.S.); isabel.k.schneider@fau.de (K.I.S.); bernardo.reyesmedina@klinikum-bayreuth.de (B.R.M.); fatemeh.khafaji@klinikum-bayreuth.de (F.K.); safwan.saffour@klinikum-bayreuth.de (S.S.); stefan.linsler@klinikum-bayreuth.de (S.L.); 2 Institute of Neuropathology, Saarland University, Medical Campus Homburg/Saar, 66421 Homburg, Germany; walter.schulz-schaeffer@uks.eu; 3 Refined Laser Systems GmbH, 48149 Münster, Germany; brinkmann@refined-lasers.com (M.B.); neumann@refined-lasers.com (F.N.); droop@refined-lasers.com (R.D.); ullmann@refined-lasers.com (S.U.); wuerthwein@refined-lasers.com (T.W.); hellwig@refined-lasers.com (T.H.); 4 Institute of Neuropathology, Friedrich Alexander University, 91054 Erlangen, Germany; lucas.hoffmann@uk-erlangen.de; 5 Department of Neurosurgery, Saarland University, Medical Campus Homburg/Saar, 66421 Homburg, Germany; nathan.monfroy@uks.eu

**Keywords:** glioblastoma, Raman, spectroscopy, histology

## Abstract

Background: Glioblastoma (GBM) remains the most aggressive primary brain tumor, and intraoperative frozen section analysis is the current standard for rapid histopathological assessment. However, this approach is time-consuming and resource-intensive. Stimulated Raman scattering (SRS) imaging has emerged as a label-free technique enabling near real-time microscopic evaluation of fresh tissue. This study compares the visualization of selected histopathological features in a newly developed intraoperative SRS system with conventional hematoxylin–eosin (HE) staining in confirmed GBM. Methods: Tumor samples from 30 patients with neuropathologically confirmed GBM were analyzed. For each case, both HE-stained frozen sections and SRS-generated virtual HE-like images were prepared from separate portions of the specimen. Twelve neuropathologists with varying levels of experience assessed 60 images according to seven predefined GBM criteria, resulting in 720 image evaluations. Feature detection was analyzed using cluster-adjusted generalized estimating equation models, and interobserver agreement was assessed using Fleiss’ κ. Results: Descriptively, hypercellularity and hypervascularization were identified at similar frequencies in both modalities, whereas pleomorphism, endothelial proliferation, mitotic activity, and necrosis were more often recognized in HE images. In cluster-adjusted analyses, SRS showed significantly lower detection rates for hypercellularity, pleomorphism, endothelial proliferation, and mitotic activity, while no significant difference was observed for hypervascularization, necrosis, or pseudopalisading after false discovery rate correction. Interobserver agreement was feature-dependent and generally higher for HE than SRS, particularly for hypercellularity. Conclusions: In this feature-level analysis of neuropathologically confirmed GBM, SRS imaging provided rapid, label-free morphological information and showed comparable visualization of selected histopathological features, particularly hypervascularization. While conventional HE-stained frozen sections remained superior for certain WHO-defining features, SRS represents a promising intraoperative adjunct that may complement established neuropathological workflows. Further studies including non-tumor tissue and a broader range of glioma grades are needed to determine the full diagnostic accuracy and clinical applicability of this technique.

## Simple Summary

Glioblastoma is a very aggressive brain tumor that must be identified quickly during surgery to guide treatment. Currently, doctors rely on frozen tissue sections stained in the laboratory, a process that takes time and requires specialized resources. This study explores whether a newer imaging method, called stimulated Raman scattering (SRS) imaging, can provide similar diagnostic information more quickly and without chemical staining. The authors compared SRS images with standard microscope slides from 30 patients and asked expert brain pathologists to evaluate both. The results show that SRS can reliably detect several key tumor features and performs nearly as well as the standard method. These findings suggest that SRS imaging could become a faster and simpler tool during brain surgery, helping surgeons to make decisions in real time and potentially improving patient care while reducing workload in pathology labs.

## 1. Introduction

Glioblastoma is the most frequent and most aggressive malignant brain tumor, associated with a dismal prognosis [1]. Standard therapy comprises surgical resection followed by concomitant radio-chemotherapy and, in some cases, additional adjuvant modalities such as tumor-treating fields [2,3].

During surgery, intraoperative frozen sections are routinely prepared to provide a preliminary pathological diagnosis and, in some cases, to assess the resection margin for residual tumor tissue [4]. For this purpose, a specimen of tumor tissue is rapidly frozen, stained with hematoxylin and eosin (HE), and examined microscopically—a process that demands time, personnel, and laboratory resources. Stimulated Raman scattering (SRS) imaging has recently emerged as a powerful, label-free method for real-time pathological assessment of fresh tissue [5]. The technique is based on the Raman effect, first described by C. V. Raman, who demonstrated that the interaction of light with matter can lead to inelastic scattering, in which photons gain or lose energy [6]. SRS exploits this effect to detect intrinsic molecular vibrations, enabling detailed microscopic visualization without staining. Analysis of Raman signals within the CH-stretch region (2800–3300 cm^−1^) allows clear differentiation between cell nuclei and cytoplasm—two features traditionally highlighted by HE staining [7,8]. Using this approach, label-free “virtual HE” images have been shown to closely replicate conventional HE-stained histology across various tissue types, including brain, gastric, prostate, and breast tumors [7,8,9].

To meet the need for rapid intraoperative microscopic imaging, a novel SRS device has been developed that enables “virtual” HE-like sections directly at the bedside [10]. This portable system is designed for use in the operating theater, for example during neurosurgical tumor resections. Previous studies have demonstrated the feasibility of stimulated Raman histology (SRH) for rapid intraoperative evaluation of brain tumors. For example, Di et al. reported that SRH can provide histology-like images enabling intraoperative interpretation of gliomas with diagnostic performance approaching that of conventional frozen section analysis [11]. While these studies primarily focused on overall diagnostic feasibility, the present study evaluates the reliability of SRS imaging at the level of individual histopathological features in confirmed glioblastoma specimens. The central question of the present study is therefore whether images generated by this device provide comparable quality—in terms of histopathological assessment, tumor representation, and interobserver reliability—to conventional HE-stained frozen sections in patients with glioblastoma.

## 2. Methods

### 2.1. Patients

The study design was informed by STARD recommendations where applicable [12]. Tumor tissues from 30 patients with radiographic findings consistent with GBM and subsequent histopathological confirmation were included. All patients underwent surgery at the Department of Neurosurgery, Medical Campus Upper Franconia/Bayreuth, Germany, between 2024 and 2025.

Inclusion criteria were: neuropathologically confirmed GBM diagnosis, sufficient tumor material for further analysis, and written informed consent. Measurements were performed on ex-vivo surplus tissue after clinical procedures and did not influence patient care. The study was approved by the local ethics committee (Ethikkommission der Friedrich-Alexander-Universität Erlangen/UKE, Germany; approval number 24-116B).

### 2.2. Study Design

For each of the 30 GBM cases, the specimen was divided into two parts and one part was used to generate a conventional HE-stained frozen section and the other to generate a “virtual” Raman imaging-based section, resulting in a dataset of 60 histological images. To assess image quality, a structured questionnaire comprising seven key neuropathological criteria of GBM was developed (Table 1). In a multi-center approach, the questionnaire was completed by 12 neuropathologists at various stages of training from the Institutes of Neuropathology at the University Hospitals of Homburg/Saar (UKS) and University Hospitals of Erlangen (FAU), as well as the Medical Campus Upper Franconia/Bayreuth. Before the study, none of the participating neuropathologists had prior experience with SRS imaging. In total, 720 individual GBM image evaluations were performed, yielding 5760 specific micromorphological assessments.

### 2.3. HE Staining

Tumor tissue was frozen and sectioned at 7 µm thickness in a cryostat. The sections were briefly dried, fixed in paraformaldehyde for one minute, and washed. Slides were immersed in hematoxylin for 60 s, washed again, then stained with eosin for approximately 30 s and rinsed. The sections were subsequently dehydrated in a graded ethanol series (70%, 80%, 90%, 100%), cleared in xylene, and coverslipped. Images were captured using a Leica DM500B microscope equipped with a Leica DFC420C camera system (Leica Microsystems, Wetzlar, Germany).

### 2.4. Raman Imaging

**Figure 1:**
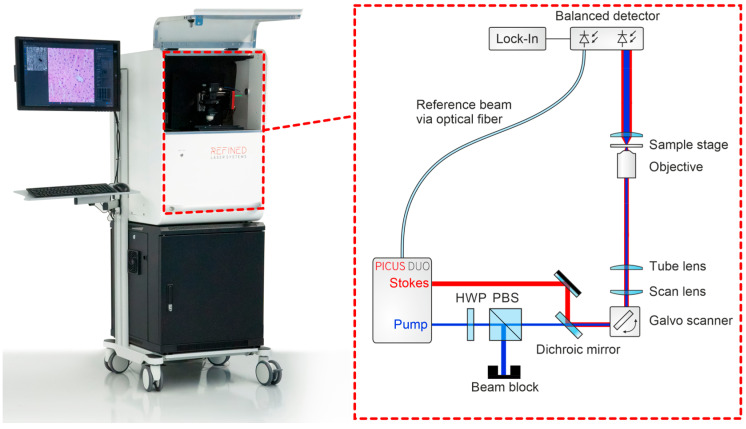
Clinical setup of the SRS imaging system. Illustration of the compact, mobile trolley integrating the laser module, detection unit, and microscope for intraoperative use. The portable intraoperative imaging system integrates a fiber-based picosecond laser source (Picus Duo, Refined Laser Systems GmbH, Münster, Germany), galvanometric scanners (Scannermax Saturn 5B, Edmund Optics GmbH, Mainz, Germany), a microscope imaging module equipped with a Nikon CFI Plan Apochromat Lambda D 20× objective (Nikon Corporation, Tokyo, Japan), and a balanced detection unit coupled to a lock-in amplifier (HF2LI, Zurich Instruments AG, Zurich, Switzerland). Data acquisition and system control were performed using a National Instruments PCIe-6361 DAQ card (National Instruments Corp., Austin, TX, USA).

**Figure 2:**
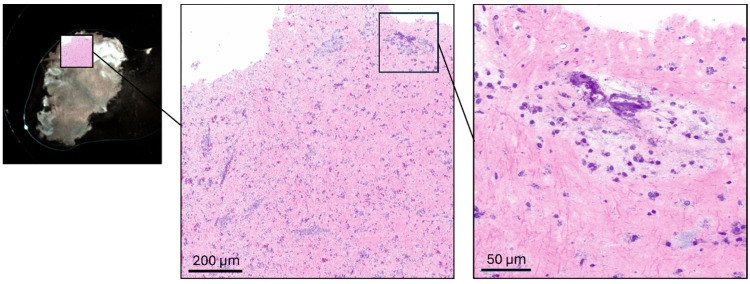
(**Left side**) Initial macro-overview image of the specimen acquired with a white-light camera (U3-38LxXLS-C, IDS Imaging Development Systems GmbH, Obersulm, Germany) for orientation. (**Middle**) Rapid low-resolution SRS scan used to identify a representative region of interest (ROI) within the tissue sample. (**Right side**) High-resolution SRS imaging of the selected ROI for detailed cellular and histomorphological assessment.

The mobile prototype imaging system contained a pair of galvanometric scanners (Scannermax Saturn 5B, Edmund Optics GmbH, Mainz, Germany) to raster scan a laser beam over a sample. The system is based on a compact, portable laser source (Picus Duo, Refined Laser Systems GmbH, Münster, Germany) coupled with a custom-designed low-noise detector. The all-fiber light source is tunable within milliseconds across 700–3100 cm^−1^, covering the fingerprint, silent, and high-wavenumber regions [13]. The air-cooled laser module (45 × 45 cm^2^) allows mobile operation in clinical settings. The robust fiber design enables integration into a wheeled, encapsulated clinical trolley (Figure 1). For SRS imaging in the CH-stretch region, images used for virtual HE rendering were acquired at 2850 cm^−1^ and 2940 cm^−1^, enabling contrast between lipid-rich cytoplasmic structures and protein-rich cellular components. In addition, spectral acquisition in the fingerprint region was performed from 750 to 1750 cm^−1^ in 5 cm^−1^ steps, resulting in 201 spectral sampling points. Excess laser noise is suppressed by dual balanced photodetectors, where a reference beam signal is subtracted post-sample, reducing common-mode noise and improving the signal-to-noise ratio by up to tenfold. SRS signals were detected using a balanced detector system (Refined Laser Systems GmbH, Münster, Germany) in combination with a lock-in amplifier (HF2LI, Zurich Instruments AG, Zurich, Switzerland). System control and data acquisition were performed using a data acquisition card (PCIe-6361, National Instruments Corp., Austin, TX, USA). Laser power at the focal plane was approximately 175 mW for the Stokes beam and 40 mW for the pump beam, with variations of approximately ±10% across the spectral tuning range. The excitation beam was focused onto the specimen using a microscope objective (CFI Plan Apochromat Lambda D 20×, Nikon Corporation, Tokyo, Japan), and transmitted light was collected using a condenser (Abbe condenser NA 0.90, Nikon Corporation, Tokyo, Japan). For imaging, a tumor specimen (~8 mm^3^) was placed on a microscope slide with a 0.1 mm spacer and covered with a coverslip. Pixel dwell time was 40 µs for CH-stretch imaging and 10 µs for fingerprint spectral acquisition. The resulting pixel size was approximately 0.25 µm for virtual HE imaging and 1 µm for fingerprint spectral scans. Typical acquisition times ranged from 30 to 60 s for overview scans, approximately 2 min for high-resolution virtual HE imaging, and up to 8 min for fingerprint spectral acquisition. Low- and high-magnification SRS modes allowed region-of-interest detection and detailed cellular visualization. Widefield white-light imaging provided structural guidance during SRS acquisition (Figure 2). The macro-overview image was acquired using a camera (U3-38LxXLS-C, IDS Imaging Development Systems GmbH, Obersulm, Germany) mounted adjacent to the microscope objective. To facilitate interpretation by neuropathologists, grayscale SRS intensity distributions were converted into pseudo-colored “virtual HE” images. Two SRS images were acquired at 2850 cm^−1^ and 2940 cm^−1^, and the nuclear signal was isolated by channel subtraction (SRS_nuclei_ = SRS_2940_ − SRS_2850_). The lipid-rich cytoplasmic signal (2850 cm^−1^) was mapped to an eosin-like pink color, while the nuclear signal was mapped to a hematoxylin-like purple tone using predefined lookup tables. The two colored channels were subsequently overlaid to generate the final virtual histology image resembling conventional HE staining, following previously described stimulated Raman histology approaches [7,8].

For SRS imaging, a region of interest (ROI) was selected based on the initial widefield overview image to identify representative tumor areas within the specimen. ROI selection was performed by the operator to capture morphologically representative regions of the tumor tissue. Because SRS imaging and HE sectioning were performed on separate portions of the specimen, the resulting images represent independent regions of interest rather than co-registered fields. This approach reflects the practical intraoperative workflow, where different parts of the specimen may be used for optical imaging and frozen section preparation.

Images were presented to the examiners in a randomized order to minimize systematic bias. The raters were blinded to the true imaging modality and evaluated each image independently using the predefined questionnaire. No dedicated training session or example images were provided prior to the evaluation in order to capture the observers’ unbiased interpretation of SRS morphology. Each image was assessed only once per examiner, and therefore no washout period was required.

### 2.5. Statistics

IBM SPSS Statistics (version 23; IBM Corp., Armonk, NY, USA) was used for statistics. Statistical analyses were performed using generalized estimating equation (GEE) models with binomial distribution to account for the clustered structure of the data, as each image was evaluated by multiple examiners. Feature detection (binary outcome) was modeled with imaging modality (SRS vs. HE) as the main predictor, and clustering was specified at the image level (Bild_ID). Effect sizes were reported as odds ratios (ORs) with 95% confidence intervals. To assess the robustness of the findings, a sensitivity analysis was performed using examiner-level clustering (Examiner_ID). Interobserver agreement was assessed using Fleiss’ κ for each histopathological feature and imaging modality separately. To control for multiple comparisons across the evaluated features, *p*-values were adjusted using the Benjamini–Hochberg false discovery rate (FDR) procedure. Statistical significance was defined as *p* < 0.05.

## 3. Results

### 3.1. Descriptive Data

**Figure 3:**
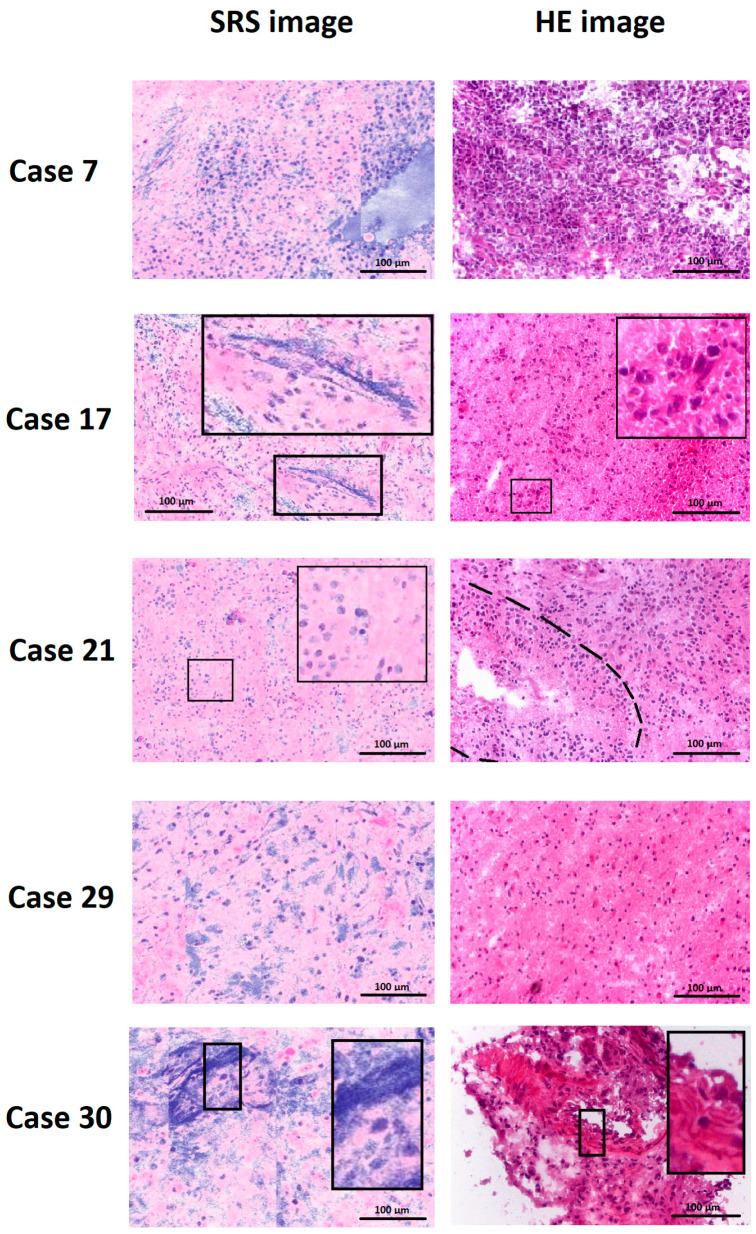
Representative examples of SRS and HE images from the study dataset.

The 30 included patients had a mean age of 65.4 ± 11.1 years [47.2–83.8]. The male-to-female ratio was 2.75:1. Gross total or subtotal tumor resection was achieved in all cases. Postoperatively, all patients received concomitant radio-chemotherapy. As of October 2025, 10 of 30 (33.3%) patients were alive. Mean overall survival was 10.4 ± 8.1 months [0.5–21.2]. Each of the 30 GBM cases yielded one Raman and one HE image, which were evaluated by 12 neuropathologists or residents, resulting in 360 Raman and 360 HE image assessments. Examiners included six residents (50%), four consultants (33.3%), and two department heads (16.7%), with a mean of 10 ± 10 years of neuropathological experience [1–31]. Assessed features included hypercellularity, cellular and nuclear pleomorphism, hypervascularization, endothelial proliferation, increased mitotic activity, necrosis, pseudopalisading, and recognition of the imaging modality (Raman vs. HE). Representative images are illustrated in Figure 3.

Left panels show SRS-generated “virtual HE-like” images, while right panels display conventional HE-stained frozen sections obtained from the same tumor specimens. Because SRS and HE imaging were performed on independent regions of interest within each specimen, the panels illustrate representative morphology rather than exact field-to-field correspondence. Characteristic glioblastoma features are highlighted. Cases 7 and 29 demonstrate hypercellularity in both imaging modalities. In case 17, the SRS image highlights necrosis in the inset, while the right hand side HE image illustrates mitotic figures. Similarly, the SRS image of case 21 demonstrates a mitotic figure in the inset, whereas the HE image of the same case shows pseudopalisading around necrosis, indicated by the dashed line. Case 30 illustrates endothelial proliferation in the insets of both modalities.

**Figure 4:**
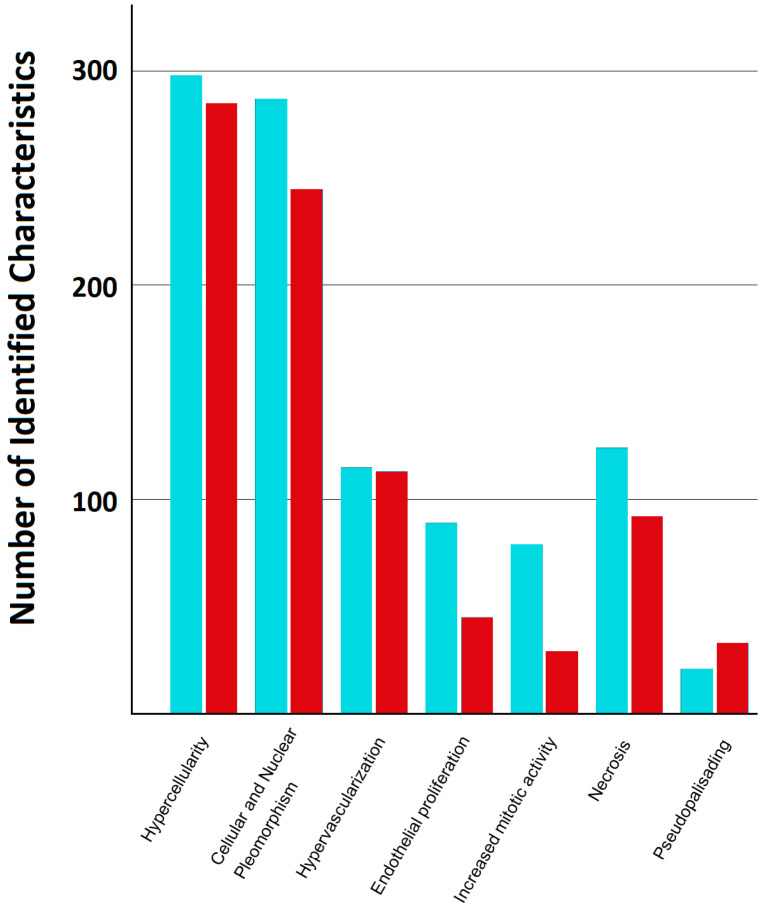
Comparative assessment of microscopic GBM features. The *x*-axis lists the seven evaluated histopathological characteristics; the bars represent descriptive counts of feature recognition across modalities. Turquoise bars represent HE-stained images, and red bars represent SRS-generated images. Statistical comparison between modalities was performed using cluster-adjusted GEE models (see Table 2).

Hypercellularity was identified in 298/360 (82.8%) HE and 285/360 (79.2%) Raman images. Cellular and nuclear pleomorphism was recognized in 287/360 (79.8%) HE vs. 245/360 (68.1%) Raman images. Hypervascularization was seen in 115/360 (31.9%) HE vs. 113/360 (31.4%) Raman images; endothelial proliferation in 89/360 (24.7%) HE vs. 45/360 (12.5%) Raman; increased mitotic activity in 79/360 (21.9%) HE vs. 29/360 (8.1%) Raman; necrosis in 124/360 (34.4%) HE vs. 92/360 (25.6%) Raman; and pseudopalisading in 21/360 (5.8%) HE vs. 33/360 (9.2%) Raman images. Of the HE images, 341/360 (94.7%) were correctly identified as such, compared to 306/360 (85%) of the Raman images (Figure 4).

The descriptive frequencies illustrate how often individual histopathological features were recognized in HE and SRS images. However, since each image was evaluated by multiple examiners, these observations are clustered and therefore not statistically independent. To account for this hierarchical structure, modality effects were analyzed using generalized estimating equation (GEE) models with image-level clustering. Odds ratios (ORs) with 95% confidence intervals (CIs) are reported for SRS compared to HE. *p*-values were adjusted for multiple comparisons across the seven features using the Benjamini–Hochberg false discovery rate (FDR) procedure. Details are highlighted in Table 2. Thus, detection rates for hypercellularity, pleomorphism, endothelial proliferation, and mitotic activity were significantly lower in SRS compared to HE after correction for multiple testing. No significant difference was observed for hypervascularization, necrosis, or pseudopalisading following FDR adjustment. Sensitivity analysis using examiner-level clustering yielded directionally consistent results, supporting robustness of the modality effects.

### 3.2. Exploratory Feature Visibility

To explore patterns of feature visibility across images, we defined evaluations in which ≥3 GBM features were identifiable as “good evaluations”. Because each image was rated by 12 examiners, images receiving ≥6 of such evaluations were categorized as “good images”. These thresholds were used for exploratory descriptive analysis only and were not intended as a formal measure of image quality. We acknowledge that this construct may be influenced by biological heterogeneity within tumor tissue, and therefore the results are interpreted as descriptive rather than inferential.

Using this definition, 22/30 (73.3%) HE images and 17/30 (56.6%) SRS images were rated as good. As SRS and H&E were acquired from independent Regions of interest (ROIs), the good image percentage is sensitive to between-field biological heterogeneity (e.g., tumor cellularity, necrosis) and thus conflates image quality with ROI content. Accordingly, we report good image counts and percentages as exploratory, descriptive patterns only.

### 3.3. Interobserver Agreement

Interobserver agreement was quantified using Fleiss’ κ separately for each feature and imaging modality (bootstrap 95% confidence intervals across images). Details can be found in Table 3. Agreement levels were strongly feature-dependent and generally higher for HE than SRS in features with higher prevalence, particularly hypercellularity. For low-prevalence features such as mitotic activity and pseudopalisading, agreement was low in both modalities.

### 3.4. Examiner Proficiency

To explore the potential influence of examiner experience, descriptive analyses were repeated for consultant-level examiners only (n = 6; 180 HE and 180 SRS assessments). The overall pattern of feature recognition was broadly consistent with that observed in the full cohort. Because this subgroup analysis was exploratory and the sample size was limited, no formal statistical comparisons between modalities were performed. Descriptive results are summarized in Table 4.

## 4. Discussion

This study investigated whether SRS-generated microscopic images of GBM specimens provide comparable diagnostic quality to conventional HE-stained sections in terms of feature recognition. SRS imaging showed comparable performance for selected features. However, for cellular and nuclear pleomorphism, endothelial proliferation, mitotic activity, and necrosis, HE staining remained superior.

The potential of SRS for rapid intraoperative imaging extends beyond neuro-oncology. High specificity rates have also been reported for otolaryngological and colorectal tumors as well [14,15]. Nevertheless, most recent advances have occurred in neuro-oncology, as highlighted by the review of Stupak et al. [16]. Early experimental studies using rat C6 glioma models demonstrated good differentiation between tumor and healthy tissue [17]. Later investigations confirmed that SRS can distinguish gliomas, metastases, meningiomas, pituitary adenomas, necrosis, and normal brain tissue with sensitivities ranging from 33 to 97%, depending on tumor type. While SRS image quality was generally high, it was occasionally inferior to HE frozen sections; in one study, 78% of cases were correctly identified as having a tumor in SRS versus 94% in HE [18]. Previous work has also demonstrated the feasibility of stimulated Raman histology for intraoperative glioma diagnosis. For example, Di et al. reported that SRH can generate histology-like images enabling rapid intraoperative interpretation of gliomas with diagnostic performance approaching that of conventional frozen section analysis [11]. The findings of the present study complement these results by focusing on the reliability of identifying individual histopathological features of glioblastoma in SRS images. By systematically evaluating feature recognition and interobserver agreement, our results provide additional insight into how SRS images are interpreted at the microscopic level.

The primary novelty of this study, compared to prior work, lies in its systematic evaluation of individual microscopic diagnostic criteria in glioma. Unlike previous studies that primarily assessed the ability of SRS to distinguish between broad tumor entities, we for the first time investigated the reliability and interobserver agreement of SRS in identifying specific, feature-level histopathological characteristics, such as hypercellularity, pleomorphism, and pseudopalisading. By combining this detailed, feature-based analysis with a multi-examiner, interobserver validation approach in a clinically relevant patient cohort, the study provides additional evidence for the potential applicability of SRS as an intraoperative adjunct in neuropathological assessment.

A major advantage of SRS is speed—both in image acquisition and evaluation—when compared to standard frozen-section workflows [19,20]. Furthermore, combining SRS with artificial intelligence (AI) has shown promising results for rapid and automated diagnosis, as demonstrated by Hollon et al. [21]. However, this benefit is not unique to SRS and also applies to digitized HE images [22].

An important consideration for intraoperative optical imaging techniques is the potential effect of laser irradiation on biological tissue. In SRS imaging, tightly focused picosecond laser pulses with milliwatt-level power are used to excite molecular vibrations. At the power levels typically employed for SRS microscopy, the energy deposition is low and predominantly non-ionizing, and previous studies have demonstrated that such irradiation does not produce detectable structural damage to biological tissues during imaging [23,24]. Moreover, acquisition times per field are short, further limiting potential thermal accumulation.

A key challenge across the literature is the lack of standardization in SRS imaging protocols. Some studies analyzed squeezed tissue samples, while others used frozen sections [25,26]. Spectral ranges used for SRS imaging vary widely, ranging from the phenylalanine band (~1005 cm^−1^) to CH-stretch regions above 3000 cm^−1^ [7,27,28]. These methodological differences complicate direct comparison of results. In our study, HE sections were cryo-cut, while SRS samples were obtained by compression, which may alter cytoarchitecture and reduce structural clarity [29]. Yet, this trade-off is essential for maintaining the intraoperative speed advantage of SRS and thus represents an inherent limitation of the technique. It is also noteworthy that even HE images displayed heterogeneity in interobserver agreement for certain features, occasionally reaching levels comparable to SRS. This indicates that SRS may achieve diagnostic reliability similar to that of conventional histology in selected parameters—an observation consistent with prior findings [30]. The next logical step will be the development of standardized and optimized protocols for SRS image generation with optimized ROI selection, potentially establishing a clinical benchmark for rapid intraoperative histopathology [16].

Several limitations should be acknowledged. First, SRS and HE images were acquired from independent regions of interest within the same tumor specimen. This may introduce variability related to intratumoral heterogeneity and precludes direct field-to-field comparison between modalities. Second, although the study incorporated a multi-rater design including examiners with different levels of neuropathological experience, the cohort consisted exclusively of neuropathologically confirmed glioblastoma cases. The present study was therefore not designed as a diagnostic accuracy study across a spectrum of tumor and non-tumor entities but rather as a feature-level agreement analysis in confirmed GBM. Consequently, the results primarily reflect the reliability of identifying individual histopathological characteristics in SRS images rather than the diagnostic accuracy of SRS for tumor classification. In addition, the exploratory classification of “good evaluations” and “good images” based on the number of identifiable histopathological features represents a pragmatic descriptive approach and may be influenced by biological variability and the presence or absence of specific tumor characteristics within a given region of interest.

Future studies including non-tumor tissue and a broader spectrum of glioma grades will be required to evaluate the full diagnostic performance and clinical applicability of this technique. Finally, while the portable SRS system enables rapid intraoperative image acquisition, further technical optimization and standardized acquisition protocols may improve visualization of specific features such as mitotic figures and microvascular proliferation. Beyond serving as an alternative to frozen sections, SRS holds promise for molecular diagnostics. While current applications focus on contrast enhancement between the nuclei and cytoplasm [7,8], in theory, the entire Raman spectrum can be exploited to extract biochemical information, such as MGMT methylation or IDH mutation status [31,32]. Early work by Liu et al. demonstrated the feasibility of using specific Raman wavenumbers combined with AI to infer molecular features from glioma architecture [33]. The device used in our study enables full-spectrum acquisition rather than narrowband detection [10,13], offering the potential to capture rich molecular information from tissue. A representative SRS spectrum acquired from glioblastoma tissue using the system is shown in Supplementary Figure S1. Such advancements could lead to a protocol with high diagnostic specificity and sensitivity, ultimately improving patient care.

## 5. Conclusions

Glioblastoma is the most aggressive primary brain tumor and requires rapid and reliable intraoperative assessment to guide surgical decision-making. The current standard, intraoperative frozen section analysis with hematoxylin–eosin (HE) staining, is accurate but time-consuming and resource-intensive. Stimulated Raman scattering (SRS) imaging is a label-free optical technique capable of generating “virtual HE-like” images from fresh tissue in near real time. In this multicenter study, tumor samples from 30 patients with neuropathologically confirmed glioblastoma were analyzed using both conventional HE frozen sections and a newly developed mobile intraoperative SRS system. Sixty images were independently evaluated by twelve neuropathologists using predefined histopathological criteria, enabling a systematic comparison of feature recognition, image quality, and interobserver agreement. While conventional HE-stained frozen sections remained superior for identifying certain WHO-defining features of glioblastoma, such as microvascular proliferation and necrosis, SRS imaging demonstrated comparable visualization of several key morphological characteristics, including hypercellularity and hypervascularization. These findings suggest that SRS can provide rapid, label-free morphological information with acceptable interobserver agreement. Within the scope of this feature-level analysis in neuropathologically confirmed glioblastoma cases, SRS imaging represents a promising intraoperative adjunct that may complement established neuropathological workflows by facilitating timely histomorphological assessment. Further studies incorporating non-tumor tissue and a broader spectrum of glioma grades are required to determine the diagnostic accuracy and full clinical applicability of this technique.

## Figures and Tables

**Table 1:** Histopathological features evaluated in the study and their operational definitions.

Feature	Operational Definition
Hypercellularity	Increased density of tumor cells compared with normal brain parenchyma
Cellular and nuclear pleomorphism	Variation in cell and nuclear size and shape typical of high-grade glioma
Hypervascularization	Increased number of blood vessels within the tumor tissue
Endothelial proliferation	Multilayered endothelial cell growth within vascular structures
Increased mitotic activity	Presence of mitotic figures indicating active cell division
Necrosis	Areas of tissue breakdown with loss of cellular structure
Pseudopalisading	Radial arrangement of tumor cells surrounding necrotic areas

**Table 2:** Cluster-adjusted comparison of feature detection (GEE analysis). Modality effect (SRS vs. HE), clustering by image (Bild_ID), and FDR-adjusted *p*-values.

Feature	OR (SRS vs. HE)	95% CI	FDR-Adjusted *p*
Hypercellularity	0.10	0.01–0.69	0.035
Cellular & nuclear pleomorphism	0.40	0.20–0.80	0.023
Hypervascularization	1.00	0.62–1.61	0.998
Endothelial proliferation	0.43	0.26–0.73	0.006
Increased mitotic activity	0.31	0.20–0.48	<0.001
Necrosis	0.58	0.32–1.04	0.079
Pseudopalisading	2.66	1.03–6.87	0.060

**Table 3:** Interobserver agreement (Fleiss’ κ) by feature and modality.

Feature	HE κ	95% CI (HE)	SRS κ	95% CI (SRS)
Hypercellularity	0.69	0.43–0.82	0.19	0.08–0.29
Cellular & nuclear pleomorphism	0.28	0.08–0.45	0.11	0.04–0.17
Hypervascularization	0.11	0.01–0.19	0.03	−0.03–0.10
Endothelial proliferation	0.11	0.03–0.18	0.04	−0.00–0.09
Increased mitotic activity	0.03	−0.02–0.08	−0.03	−0.05–0.01
Necrosis	0.14	0.05–0.20	0.05	−0.02–0.12
Pseudopalisading	0.18	−0.03–0.29	0.03	−0.02–0.08

**Table 4:** Exploratory feature recognition by consultant-level examiners.

		Recognition in 360 Assessments
	Modality	N
Hypercellularity	HE	145/180
	SRS	139/180
Cellular and Nuclear Pleomorphism	HE	138/180
	SRS	119/180
Hypervascularization	HE	44/180
	SRS	51/180
Endothelial Proliferation	HE	25/180
	SRS	11/180
Increased mitotic Activity	HE	12/180
	SRS	1/180
Necrosis	HE	35/180
	SRS	30/180
Pseudopalisading	HE	8/180
	SRS	15/180

## Data Availability

The dataset supporting the findings of this study has been made publicly available at Zenodo (DOI: 10.5281/zenodo.18908950). The dataset contains the anonymized evaluation data used for the statistical analyses reported in this manuscript.

## Supplementary Materials

The following supporting information can be downloaded at: https://www.mdpi.com/article/10.3390/cancers18071053/s1, Supplementary Figure S1. Representative stimulated Raman scattering (SRS) spectrum acquired from glioblastoma tissue using the intraoperative imaging system employed in this study. The spectrum illustrates characteristic Raman bands within the fingerprint region. Spectra derived from nuclear and cytoplasmic regions demonstrate differences in biochemical composition, including protein-associated and lipid-associated signals. These data illustrate the full spectral acquisition capability of the system.
