# Supplementary material for: Stimulated Raman Spectroscopy for Intraoperative Glioblastoma Diagnosis—A Complementary Tool to Frozen Section?
Source: Cancers (Basel). 2026 Mar 24;18(7):1053. doi: 10.3390/cancers18071053 (PMC13072363; doi:10.3390/cancers18071053)
Supplement: Supplementary file 1 [file cancers-18-01053-s001.zip › cancers-4140602-supplementary.pdf]

Supplements:

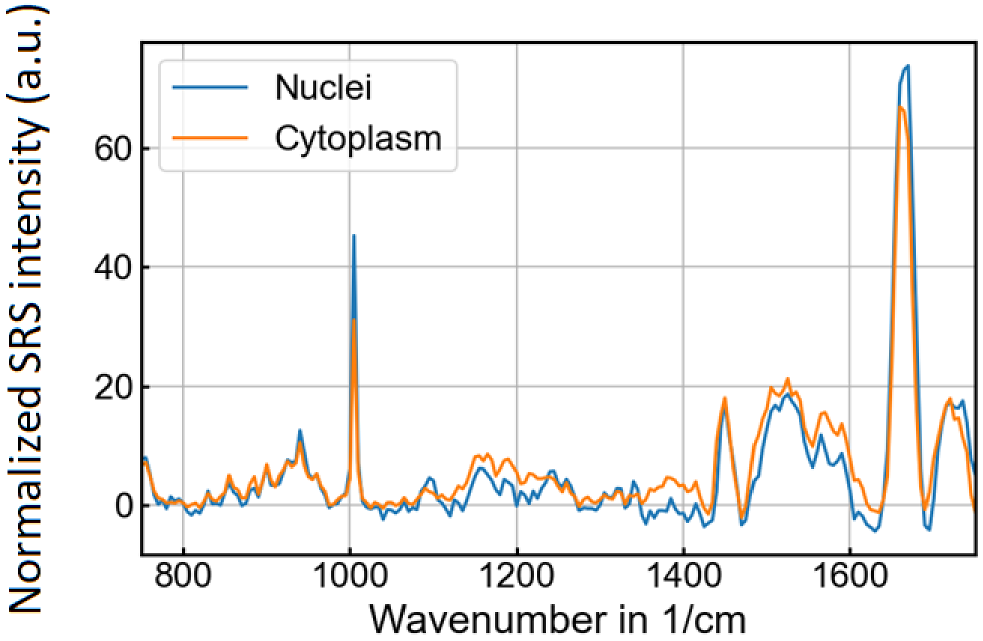

Supplementary Figure S1. Representative stimulated Raman scattering (SRS) spectrum acquired from glioblastoma tissue using the intraoperative imaging system employed in this study. The spectrum illustrates characteristic Raman bands within the fingerprint region. Spectra derived from nuclear and cytoplasmic regions demonstrate differences in biochemical composition, including protein-associated and lipid-associated signals. These data illustrate the full spectral acquisition capability of the system.
